# Gradient-Based Radiomics for Outcome Prediction and Decision-Making in PULSAR: A Preliminary Study

**DOI:** 10.1016/j.ijpt.2025.100739

**Published:** 2025-02-03

**Authors:** Haozhao Zhang, Jiaqi Liu, Michael Dohopolski, Zabi Wardak, Robert Timmerman, Hao Peng

**Affiliations:** a Department of Radiation Oncology, The University of Texas Southwestern Medical Center, Dallas, TX 75390, USA; b Medical Artificial Intelligence and Automation Laboratory, The University of Texas Southwestern Medical Center, Dallas, TX 75390, USA

**Keywords:** Radiomics, Feature selection, PULSAR, Brain metastasis, Outcome prediction

## Abstract

**Purpose:**

Personalized ultrafractionated stereotactic adaptive radiation therapy (PULSAR) has emerged as an innovative method for delivering high-dose radiation over extended intervals, adapting treatment based on the patient’s response. However, current adaptation largely relies on physicians’ experience and tumor size assessment, underscoring the need for a data-driven approach to improve outcome prediction and support decision-making.

**Materials and Methods:**

We analyzed 69 lesions from 39 patients undergoing PULSAR treatment. Gradient-based features, including gradient magnitude, radial gradient, and radial deviation, were extracted from both intratumoral and peritumoral regions, with the latter further divided into octant subregions. Support vector machine models were developed using features from first magnetic resonance imaging (MRI), second MRI, and delta mode (change between the 2). An ensemble feature selection (EFS) model was then created by combining the features of the top-performing individual models. The approach was validated on a non-PULSAR cohort (37 lesions from 23 patients) treated with standard fractionated stereotactic radiation therapy.

**Results:**

The EFS model shows strong predictive performance in determining whether tumor volume reduction exceeds 20% at the 3-month postradiation time point. Features derived from octant subregions exhibit significantly better prediction than those from the core or entire margin. Pretreatment features (from first MRI) generally outperform second MRI and delta-mode features, while the inclusion of 1 delta feature in the EFS model enhances performance. In the non-PULSAR cohort, the gradient-based approach outperforms conventional radiomics, demonstrating its strong generalizability.

**Conclusion:**

Our gradient-based radiomics approach, combining spatial segmentation and temporal features, significantly enhances treatment response prediction in PULSAR therapy. Its superior performance compared to conventional radiomics, coupled with its effectiveness in both PULSAR and non-PULSAR cohorts, highlights its potential as a robust tool for personalized treatment planning in neuro-oncology, applicable to both photon and particle therapies.

## Introduction

**Figure 1:**
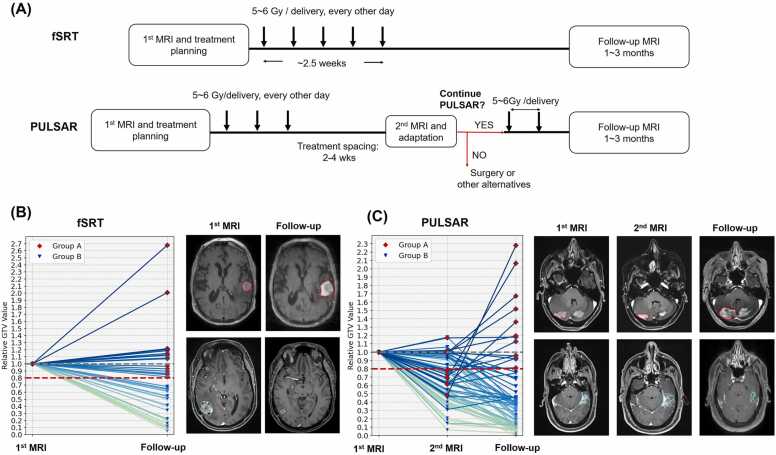
(A) Workflow comparison: fSRT uses the first and follow-up MRIs, while PULSAR adds a second MRI during treatment. (B) In fSRT, lesion volumes are assessed at 2 time points (initial and follow-up). Examples show a lesion with increased GTV post treatment (top) and one with reduced GTV (bottom). (C) In PULSAR, lesion volumes are assessed at 3-time points (first MRI, second MRI, and follow-up). Examples illustrate increased GTV after treatment (top) and reduced GTV (bottom). **Abbreviations**: PULSAR, personalized ultrafractionated stereotactic adaptive radiation therapy; fSRT, fractionated stereotactic radiation therapy; and GTV, gross tumor volume; MRI, magnetic resonance imaging.

At the University of Texas Southwestern Medical Center, we are exploring an innovative approach for managing brain metastases (BMs) called personalized ultrafractionated stereotactic adaptive radiation therapy (PULSAR).^1^, ^2^, ^3^, ^4^, ^5^ It is an advanced approach to radiation therapy, whether photon or particle therapy, which involves evaluating tumor changes during treatment to personalize and adjust radiation delivery, aiming to optimize therapeutic outcomes while minimizing side effects. PULSAR is designed to deliver high-dose radiation at 2 to 4-week intervals (Figure 1A), enabling timely intratreatment adjustments, personalized treatment planning, enhanced normal tissue recovery, and potential synergy with concurrent immunotherapeutic approaches. The selection of a 2 to 4-week interval involves multiple considerations still under investigation, including enhanced tumor downstaging, increased normal tissue sparing, alignment with typical immune drug dosing cycles, tracking tumor anatomy changes, and ensuring patient convenience. However, waiting too long is not feasible due to the risk of tumor repopulation. We anticipate that PULSAR will be particularly valuable in particle therapy due to its high precision, the sensitivity of dose distribution to small variations, and its ability to reduce overall treatment time.

A key element in maximizing PULSAR’s potential is effective decision-making both before and during treatment (Figure 1A), determining the optimal timing for adjustments and identifying the patients who will benefit most. Currently, treatment plans are usually adjusted based on changes in gross tumor volume (GTV) observed in the second magnetic resonance imaging (MRI) scan (eg, a 20% GTV change as a threshold) compared to the first MRI scan. However, this assessment relies heavily on the physician’s expertise and experience, which can introduce bias and may not always align with treatment outcomes. A more robust, data-driven approach for PULSAR treatment is therefore highly desirable.

Numerous studies have explored the analysis of pretreatment MRI images (also referred to as the first MRI in our study) to predict outcomes for BMs. Local control and failure are typically assessed using routine MRI scans that track changes in tumor size. Meanwhile, radiomics has become an essential tool for extracting quantitative features to predict disease phenotypes and treatment responses.^6^, ^7^ However, several challenges remain. Firstly, conventional radiomics methods often generate a large number of features, leading to redundancy and increased computational complexity. The inclusion of irrelevant or redundant features complicates the feature selection process and undermines the robustness and stability of the model.^8^, ^9^ Secondly, many strategies apply mathematical transformations to preprocess medical images in order to capture detailed spatial frequency information.^10^ However, these preprocessing steps can complicate interpretation. Thirdly, most radiomics studies focus primarily on intratumoral features, overlooking the potential predictive value of peritumoral features for assessing treatment response. One representative work is by Wang et al,^11^ who investigated peritumoral radiomics by expanding the GTV with a margin ranging from 5 to 20 mm and using 100 features, at the cost of a high computational burden.

The potential of nonstandard radiomics in predicting treatment response has gained increasing attention. Presented below are some notable examples, along with their limitations. Tunali et al^12^ utilized radial gradient (RG) and radial deviation (RD) features extracted from pretreatment CT images to predict lung cancer survival. While the study highlighted the correlation between gradient-based features and survival rates, the authors treated the region of interest as a single piece without considering spatial distribution.^12^ Similarly, Caballo et al^13^ developed multimarker radiomic models for lesion characterization with breast CT images, confirming the predictive value of peritumoral features. However, the study only extracted features based on 2D axial images. Hao et al^14^ introduced the shell feature for predicting distant failure in lung and cervix cancer, a PET-derived radiomics model focusing on the tumor periphery. While effective in capturing boundary heterogeneity, projecting a series of subshells across multiple slices into a 2D map results in a loss of spatial information.^14^ Additionally, all these previous studies included only a single time point, not accounting for temporal changes.

In this preliminary study, we propose utilizing gradient-based metrics for PULSAR treatment based on patient data of BMs, the most common type of intracranial tumors.^15^, ^16^, ^17^ Our proposed gradient-based metrics offer several potential advantages. Basic metrics such as mean intensity and standard deviation are simpler to compute and interpret compared to complex features. Furthermore, they are less sensitive to noise or variations in imaging acquisition protocols and parameters, ensuring greater robustness and reliability across different imaging modalities. Three gradient-based features are tested in this study, including gradient magnitude (GM), RG, and RD. These metrics capture spatial variations in tumor intensity, directional characteristics of tumor growth or shrinkage, and angular deviations of intensity changes relative to the tumor center. Partitioning the tumor area into core and subregions allows for a more detailed examination of the tumor’s spatial characteristics. Furthermore, given the unique nature of PULSAR, we have access to delta features extracted from sequential imaging (eg, first and second MRI). This enables us to evaluate whether gradient-based features at different time points provide additional insights into predicting treatment outcomes and distinguish itself from previous studies that focus solely on pretreatment data.

## Methods

### Data collection

Figure 1 depicts the workflow comparison between standard fractionated stereotactic radiation therapy (fSRT) and PULSAR for patients with BMs undergoing radiosurgery utilizing Gamma Knife Icon (Elekta AB). In the PULSAR protocol, patients first undergo a pretreatment MRI scan followed by the initial treatment course, which consists of 3 fractions or pulses (5-6 Gy per fraction/pulse) with a 2-day interval between fractions. Subsequently, after a span of 3 weeks, the second treatment cycle is administered based on the second MRI scan, allowing for adjustments to changes in tumor volume. Our retrospective study focused on 39 patients who underwent PULSAR treatment at University of Texas Southwestern Medical Center. This cohort included 69 lesions treated between November 1, 2021, and May 1, 2023, encompassing patients with both single and multiple metastases. Detailed demographic and clinical profiles, such as age, gender, histology, lesion number, and treatment specifics, are provided in Table. During the treatment process, the GTV, clinical target volume, and planning target volume were considered equivalent, as no margin expansions were applied. Lesions with a Gradient Index >4 were excluded from the study.

**Table:** Demographics and clinical profiles of patients receiving PULSAR versus fSRT.

	PULSAR cohort	fSRT cohort
Patient number	39	23
Age (range), y/o	61 (28-84)	68 (47-86)
Gender (male: female)	14:25	11:12
Initial tumor volume (mm^3^)	3666 (14.5-37,607)	3188 (15.8-33,564)
3D diameter (mm)	25.5 (3.7-79.8)	26.4 (3.7-81.5)
Lesion number	69	37
(Decreased: nondecreased^a^)	(55:14)	(19:18)
Single brain metastases	26	14
Multiple brain metastases	43	23
Dose and fraction groups (Gy, Fx)		
30 Gy, 5Fx	57	22
27.5 Gy, 5Fx	2	3
25 Gy, 5Fx	9	10
24 Gy, 4Fx	1	2
Histology		
Breast	25	6
Colorectal	4	3
Renal	4	1
Melanoma	2	1
NSCLC	24	13
GYN^b^	4	8
Other^c^	6	5

**Abbreviations: PULSAR, personalized ultrafractionated stereotactic adaptive radiation therapy; fSRT, fractionated stereotactic radiation therapy; GYN, gynecologic cancer**.

a A 20% change in gross tumor volume at the 3-month postradiation time point was used as the threshold. The incidence of more than a 20% volume is 79.7% for the PULSAR cohort and 51.4% for the fSRT cohort.

b The categories “uterine,” “cervix,” and “ovarian” were grouped under GYN.

c The categories “neuroendocrine,” “pineal,” “thymus,” “thyroid,” and “biliary” were grouped under Other.

We collected 2 MRI images and radiation therapy contour structure files (RTstructure). All MRI images were acquired using axial sequences with T1-weighted enhancement. This approach minimized potential discrepancies arising from different imaging modalities or sequences, thereby ensuring the accuracy of subsequent delta-omics calculations in the PULSAR cohort. Tumor volumes in follow-up MRI images (without enhancement) were assessed at the 3-month postradiation time point. Two board-certified radiation oncologists conducted a thorough comparison of MRI images and GTV contouring for each lesion. For the non-PULSAR cohort, gradient-related features were only extracted from the first MRI scan, since no delta mode was applicable. Lesions in each cohort were categorized as large or small based on their GTV to assess treatment efficacy and the application of different margins (see Octant segmentation and feature selection). Lesions with a GTV ≥4000 mm³ were classified as large, while those with a GTV <4000 mm³ were classified as small. The PULSAR cohort included 36 large and 33 small lesions, and the non-PULSAR cohort contained 18 large and 19 small lesions.

### Octant segmentation and feature selection

The morphological delineation of tumor boundaries plays a pivotal role in our study. The process involves generating a 3-dimensional tumor mask *M.* We first delineate the masks of the core and margin using morphological operations based on the lesion’s center of mass. The dilation/contraction of the mask is expressed as below:

(1) $$M_{\text{expanded}}=M⊕B_{d}\;M_{\text{contracted}}=M⊖B_{e}$$

**Figure 2:**
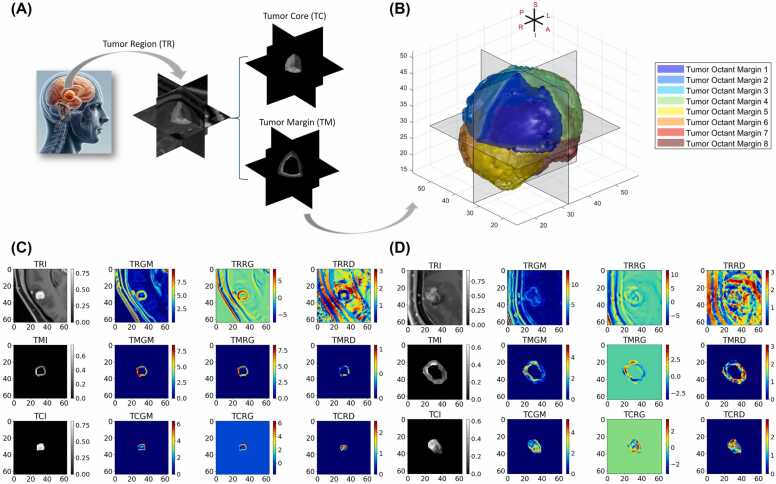
(A) Tumor core (TC) and tumor margin (TM) segmentation from the 3D tumor region (TR). (B) Orientation of the segmented tumor margin into 8 octants aligned with anatomical directions: superior (S), inferior (I), left (L), right (R), anterior (A), and posterior (P). (C) and (D) Gradient feature visualization for a small tumor (C) and a large tumor (D), with 4 columns showing the MRI image, gradient magnitude (GM), radial gradient (RG), and radial deviation (RD). Rows correspond to TR, TM, and TC.

Here, $B_{d}\;$represents the structuring element for dilation, expanding the mask by a predefined margin (3 mm for large lesions and 1 mm for small lesions). $B_{e}\;$represents the structuring element for contraction dilation, retracting the mask of the tumor core. We obtained 2 masks for subsequent analyses: core $\left(M_{\text{core}}=M_{\text{contracted}}\right)$ and margin (${M_{\text{margin}}=M}_{\text{expanded}}-M_{\text{contracted}}$). For a given point $P\left(x,y,z\right)$, it was assigned to a corresponding octant (eight in total). Ultimately, we created 10 region-specific masks: the core (*M*_*core*_), peripheral margin (*M*_*margin*_), and 8 subdivided marginal sections (*M*_*i*_, where i ranges from 1 to 8). Figure 2A and B illustrates the above process.

For a 3-dimensional image, the 3D Sobel operator was applied to calculate the spatial gradient along these axes ($G_{x},\;G_{y},G_{z}$).^18^ The 3D Sobel convolution kernels $K_{x},\;K_{y},K_{z}$ are designed to capture variations in image intensity in their respective directions. Three gradient-based features are defined in Equation (2): GM, RG, and RD:

(2) $$\mathrm{GM}=\sqrt{G_{x}^{2}+G_{y}^{2}+G_{z}^{2}}\;\vec{\mathrm{RG}}=\frac{\vec{G}\cdot \vec{D}}{\left|\vec{D}\right|}\mathrm{RD}=\arccos\left(\frac{\vec{\mathrm{RG}}}{\left|\vec{G}\right|}\right)$$

where $\vec{G}=\;\left(G_{x},\;G_{y},\;G_{z}\right)$ is the gradient vector at the voxel and $\vec{D}=\;\left(x_{\mathrm{com}}-x,\;y_{\mathrm{com}}-y,\;z_{\mathrm{com}}-z\right)$ is the radial vector from the tumor’s center of mass ($x_{\mathrm{com}}$, $y_{\mathrm{com}}$, $z_{\mathrm{om}}$) to the voxel. In brief, GM quantifies the rate of change of intensity at each voxel, with high values indicating significant changes in intensity at edges or boundaries within the image (eg, transitioning between normal brain tissue and tumor). RG and RD delve into the correlation between gradient direction at each point and the radial direction, providing further insights into spatial structures and their directional orientation. RG measures the alignment of the gradient vector with the radial direction from the center of mass to the voxel, which helps understand the directional characteristics of tumor growth or shrinkage. RD quantifies the angular deviation of the gradient vector from the radial vector.

For every mask within the 10 masks {*M*_*core*_, *M*_*margin*_, *M*_*i*_, *M*_*2*_ … *M*_*8*_}, we computed 3 gradient parameters (GM, RG, RD). The mean (*μ*) represents the average intensity, though it may not be the best measure of central tendency for asymmetric data or data with outliers. Standard deviation (*σ*) captures the variability in voxel intensities within the ROI, reflecting heterogeneity. The coefficient of variation (*CV*), the ratio of the standard deviation to the mean, provides a measure of relative variability useful for comparing regions of interest with differing sizes or intensity scales. Together, the above process yields the extraction of 90 features, derived from 3 gradient parameters, 10 masks, and 3 statistical metrics. To facilitate understanding of these features, we generated axial 2D visualizations of the tumor region, margin, and core, accompanied by their respective GM, RG, and RD maps (Figure 2C and D).

### Predictive modeling and performance evaluation

Our model is designed to predict whether a lesion will demonstrate a volume reduction of ≥ 20% at the 3-month postradiation time point. Previous studies have demonstrated a strong correlation between a volume reduction of 20% or more and improvement in neurological signs and symptoms.^19^, ^20^, ^21^ We applied the same criterion and framed it as a classification problem. Tumors with follow-up volumes at or above 80% of their initial volume were categorized as “non-responder,” while those with volumes reduced below this threshold were categorized as “responder.”

We trained multiple support vector machine (SVM) models for the classification task. Each SVM model was trained and validated using stratified k-fold cross-validation, keeping the ratio between the 2 groups constant in each fold. Performance metrics included sensitivity, specificity, accuracy, precision, F1 score, and the area under the receiver operating characteristic curve (AUC). For the pretreatment MRI data sets (first MRI), we studied 5 series: (1) core, (2) margin, (3) core/margin ratio, (4) octant margins, and (5) core+margin+octant margins, denoted by 1A to 1E, respectively. Specifically, we derived 9 features solely from the core region (1A) and solely from the entire margin (1B). Series 1C derived the ratio of features between 1A and 1B. In series 1D, we selected 9 features out of the 72 features of 8 octants (each with its own 9 features); likewise, we selected 9 features for series 1E from all 90 features (core, margin, 8 octant margins). The above analysis was then repeated based on the second MRI images (2A-2E) and the delta mode (3A-3E). In the delta mode, arithmetic operations (subtraction) were conducted on each feature obtained between the first and second MRI scans (eg, “2A-1A”) to capture the temporal changes in tumor characteristics. For series D and E, we selected 9 features from either a pool of 72 features (D series) or 90 features (E series), to uphold consistency with the number of features in series A to C. For the PULSAR cohort, a total of 15 individual models were generated. The final step was the creation of an ensemble feature selection (EFS) model, which combined features from 3 individual models (1E, 2E, and 3E) to enhance both robustness and predictive performance.

To address the challenges of a small and imbalanced PULSAR data set and to reduce the risk of overfitting, feature reduction techniques were employed (see Figure S1). The process involved 2 key steps: (1) Recursive feature elimination was performed using 5-fold stratified cross-validation over 50 iterations to identify the top 9 features in each iteration, and (2) the 9 most frequently selected features across all iterations were determined. Using these selected features, 5-fold cross-validation with 50 iterations was conducted to assess performance. In each iteration, an SVM classifier was trained on the training set and validated on the hold-out fold.

### Performance evaluation of fractionated stereotactic radiation therapy cohort

To validate our proposed approach, we conducted 2 studies for the fSRT cohort, which had a more balanced data set compared to the PULSAR cohort. The first study, referred to as the gradient model, mirrored the approach in series 1E within the PULSAR cohort. It is important to note that since only the first MRI image was available in this case, series like 2A to 2E and 3A to 3E were not applicable. Meanwhile, the follow-up MRI image sequences differed from those used in the pretreatment images, preventing a meaningful calculation of delta radiomics. In the second study, referred to as the radiomics model, we implemented conventional radiomics based on PyRadiomics^8^ and selected the top 9 features for comparison. Both the gradient model and the radiomics model were trained and validated using the same stratified k-fold cross-validation approach described in Predictive modeling and performance evaluation. The performances were assessed using the same set of evaluation metrics.

Two points require clarification. First, for the fSRT cohort (acquired during the same time period as the PULSAR cohort), several factors impacted the patient selection process, including insufficient follow-up, nonmetastatic lesions, and cases involving single-fraction treatments. As a result, only 37 lesions (Table 1) were included in this study. Second, a comprehensive evaluation of the conventional radiomics approach for the PULSAR cohort has been published recently, which motivated us to explore gradient-based features. Therefore, this manuscript does not cover standard radiomics for that cohort.^5^

## Results

### Volume change and gradient-based features

Volumetric analysis reveals a complex response to treatment across lesions. Figure 1B and C show tumor volume changes (relative to the initial GTV in the first MRI) at different time points for the fSRT and PULSAR groups, respectively. In Figure 1C, a small subset of lesions exhibits an initial increase followed by a decrease, while others show an initial decrease before increasing. These dynamics suggest that patient responses to PULSAR treatment are diverse, and evaluations using either the first or second MRI alone do not fully capture the treatment outcome,

Visualizations of tumor regions, margins, and cores are shown for a small (Figure 2C) and large lesion (Figure 2D). The tumor region image shows the tumor’s anatomical features and its relationship to surrounding structures. The tumor region gradient magnitude highlights the areas of rapid intensity change, with high gradient values observed along tumor margins and skull interfaces (eg, sharp transitions). The tumor region radial-gradient illustrates the alignment between intensity gradients and radial vectors from the tumor’s center. The tumor region radial deviation quantifies the angular difference (between 0 and π) between gradient and radial vectors, with lower values representing better alignment, typically seen in spherical tumors. In contrast, for tumors with irregular shapes, the intensity gradient is not symmetrical, leading to greater angular deviation between the gradient and radial vectors. In the second row, the margin maps (TMI, TMGM, TMRG, and TMRD) offer detailed insights into the tumor’s peripheral characteristics, while the third row shows the core maps (TCI, TCGM, TCRG, and TCRD), which reveal the internal structure and heterogeneity inside the tumor.

### Individual models

**Figure 3:**
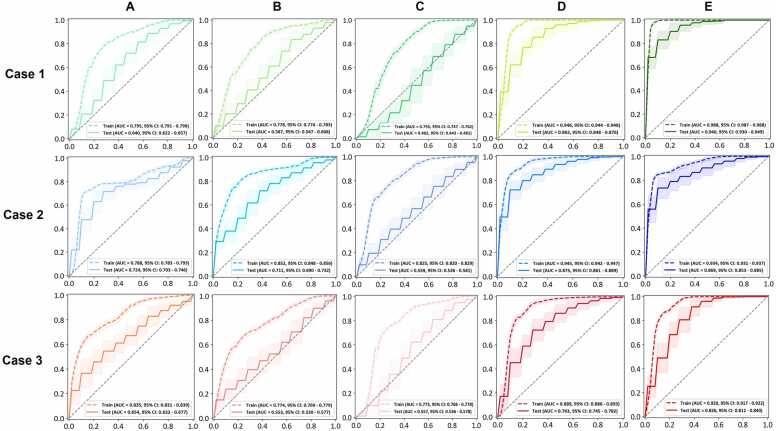
Performances of individual models derived from the PULSAR cohort. Each panel displays the receiver operating characteristic curves, with shaded areas indicating the confidence intervals (95% CI) for both training (dashed lines) and test (solid lines) sets. A total of 15 models were developed across 5 series (A-E): first MRI (1A-1E), second MRI (2A-2E), and delta mode (3A-3E). Series A-C represent models derived from features solely from the tumor core (A), tumor margin (B), and the core/margin ratio (C). (D) Models based on selected features from the 8 tumor octant margins. (E) Features from all regions (core, margin, and 8 octant margins). **Abbreviations**: PULSAR, personalized ultrafractionated stereotactic adaptive radiation therapy; AUC, area under the receiver operating characteristic curve.

The performance of individual models is summarized in Figure 3. For models in series A, B, and C, where features are extracted without segmentation into octant subregions, the AUC remains relatively low. Model 1E outperforms all other models with an average AUC exceeding 0.9, significantly higher than the others. Models 2D, 2E, and 1D show similar performance, with average AUCs around 0.8. Except for Model 1E, the other models exhibit relatively wide 95% confidence intervals. F1 scores follow a similar trend, with Model 1E achieving the highest average F1 score of approximately 0.7. Models 2D, 2E, and 1D have slightly lower F1 scores, around 0.6, while the remaining models show F1 scores below 0.55 and wide 95% confidence intervals. Detailed metrics, including sensitivity, specificity, accuracy, AUC, precision, and F1 score, along with *P*-values for paired comparisons, are presented in Tables S1 and S2.

Taking the pairwise statistical comparisons between series D and E as an example (Table S2), Series D models use the top 9 features only from octant margins, while series E models select 9 features from the core, margin, and octant margins. Models with added core and margin features generally outperform their counterparts. Model 1E outperforms Model 1D in the pretreatment mode, and Model 3E surpasses Model 3D in the delta mode. For instance, Model 3E demonstrates higher sensitivity (0.779 vs 0.674), AUC (0.826 vs 0.763), and F1 score (0.541 vs 0.483) compared to Model 3D. However, this pattern is not observed between models 2D and 2E, both of which exhibit very similar performance. Among the E series, the performance ranking is 1E > 2E > 3E. Model 1E shows higher specificity, accuracy, AUC, precision, and F1 score compared to Model 2E, with no significant difference in sensitivity.

### Ensemble feature selection model

**Figure 4:**
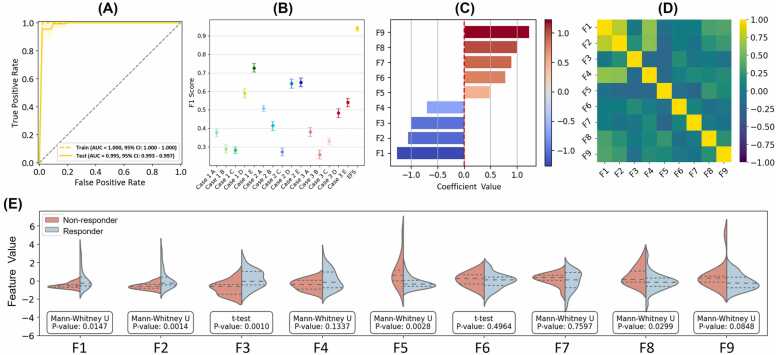
Evaluation of the ensemble feature selection (EFS) model from the personalized ultrafractionated stereotactic adaptive radiation therapy cohort. (A) Receiver operating characteristic curve showing training (dashed yellow) and test (solid yellow) performance, with AUC and 95% CI. (B) F1-score comparison highlights the EFS model’s superior performance over individual models (cases 1A-1E, 2A-2E, and 3A-3E). (C) Coefficient values of the 9 EFS-selected features, with positive values contributing to the “non-responder” group and negative values to the “responder” group. (D) Correlation heatmap of the 9 features (F1-F9), with Spearman’s correlation coefficients indicated by color intensity. (E) Violin plots of feature distributions (F1-F9) for 2 groups, with statistical significance marked by *P*-values from Mann-Whitney U or *t*-tests. Three dotted lines correspond to the 25th percentile (Q1), the median, and the 75th percentile (Q3), respectively. **Abbreviation**: AUC, area under the receiver operating characteristic curve.

The EFS model surpasses the performance of individual models by integrating the top 9 features from 3 individual models (1E, 2E, and 3E), as detailed in Tables S1 and S2, showing a sensitivity of 0.974 ± 0.104 and a specificity of 0.973 ± 0.046. Figure 4 provides a thorough performance analysis. The AUC curve indicates near-perfect performance, with the testing AUC reaching 0.995 (Figure 4A). The model achieves the highest F1 score (0.940 ± 0.103) compared to other models (Figure 4B). The coefficients of the 9 features in the SVM model vary from positive to negative (Figure 4C). Feature F9 has the highest positive coefficient (1.229), while feature F1 has the most negative coefficient (−1.261). The correlation heatmap reveals weak correlations among features, indicating low redundancy (Figure 4D). Violin plots comparing features between the "non-responder" and "responder" groups illustrate the distribution of each feature and highlight their statistical significance. Most features differ significantly between the 2 groups (*P* < .05), except for F4, F6, F7, and F9 (Figure 4E).

Table S3 summarizes the details of 9 features selected by the EFS model, all from octant margin regions (M6, M4, M1, M2, and M8). This highlights the significance of these subregions in predicting treatment response. The features encompass various gradient parameters (RG, RD, and GM) and statistical metrics (STD, mean, and CV). In Figure 4E, features with the most significant differences, indicated by their *P*-values, include F2 (*P* = .001), F3 (*P* = .001), and F5 (*P* = .003). F1 and F8 also exhibit significant differences between the 2 groups (*P* < .05). The feature weight coefficients confirm the importance of these features (F1, F2, and F3). However, different patterns are also observed. Feature F9 does not show a statistically significant difference between groups in the Mann-Whitney *U* test (*P* = .0848), yet it possesses the highest feature weight coefficient (1.229). Conversely, feature F5 exhibits a significant difference between groups (*P* = .003), but its coefficient (0.486) is lower. Such a pattern might be attributed to 2 factors. First, the limited cohort size can impact the statistical distribution of individual features, as illustrated in Figure 4E. Second, selecting features based on *P*-values does not always result in large SVM coefficients, due to feature interactions and multicollinearity.

### Nonpersonalized ultrafractionated stereotactic adaptive radiation therapy cohort

**Figure 5:**
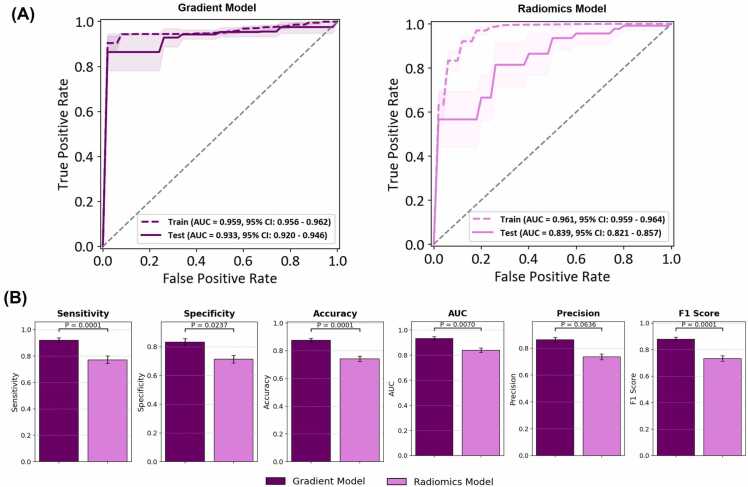
(A) Performance evaluation of the gradient and radiomics models in the fractionated stereotactic radiotherapy cohort. Receiver operating characteristic curve for the Gradient model and the Radiomics model, with AUC values and 95% CI for training (dashed line) and test (solid line) sets. (B) Comparison of predictive metrics (sensitivity, specificity, accuracy, AUC, precision, F1-score), showing the Gradient model outperforming the radiomics model in most metrics, with statistical significance indicated by *P*-values. Error bars represent standard deviations. **Abbreviations**: AUC, area under the receiver operating characteristic curve.

The results for the non-PULSAR cohort are presented in Figure 5 and Table S4. The gradient model achieves an AUC of 0.959 (95% CI: 0.956-0.962) in the training data set and 0.933 (95% CI: 0.920-0.946) in the testing data set. In contrast, the radiomics model has an AUC of 0.961 (95% CI: 0.959-0.964) in the training data set and 0.839 (95% CI: 0.821-0.857) in the testing data set. Figure 5B shows the comparison of the 6 metrics, revealing that the gradient model surpasses the radiomics model in 5 out of 6 metrics, with the exception of precision. These findings highlight the effectiveness of the proposed gradient-based framework for both PULSAR and non-PULSAR data sets, showcasing robust performance in both balanced and imbalanced scenarios.

## Discussion

This preliminary study demonstrates the feasibility of using gradient-based features for outcome prediction in BMs treated with PULSAR. We find that features extracted from segmented octant regions offer superior predictive capability compared to those derived solely from the whole core and margin, emphasizing the importance of features tied to spatial heterogeneity. Our approach has several advantages over traditional radiomics methods. Unlike conventional radiomics techniques that extract a large number of features and lead to interpretability issues, our method uses simpler, more interpretable gradient-based metrics. These gradient metrics provide directional information about tumor growth patterns and boundaries, which may be more crucial for assessing tumor heterogeneity and invasiveness. In addition, conventional features often overlook peritumoral characteristics within tumor margins. To address this, we define intratumoral (tumor core) and peritumoral (tumor margin and octant margins) regions with enhanced spatial granularity in 3 dimensions (eg, the top 4 features all come from the octant region M6 in Table S3). Furthermore, our method is able to incorporate both first and second MRI images, leveraging the unique aspect of PULSAR treatment. To assess the generalizability of our approach, we conducted a comparison between the PULSAR and non-PULSAR cohorts.

The perspective on extending treatment time intervals continues to be a subject of debate, given the complex challenge of balancing adverse effects with effective tumor control. Both PULSAR and fSRT require careful personalization, given the significant variability in individual patient responses to treatment. While some patients may experience tumor reduction, others may demonstrate tumor growth (Figure 1B and C). In addition, it is crucial to distinguish between pseudo-progression and true progression for predicting treatment outcome and guiding treatment decisions. Additional factors, such as tumor radioresistance and characteristics associated with systemic therapy, further complicate decision-making. By employing multiomics analysis and machine learning, we aim to transition the decision-making process from empirical judgments to a more data-driven approach, accurately identifying *who*, *when,* and *how* to adapt for optimizing therapeutic outcomes.

Features derived from segmented octant regions (series D/E) exhibit superior predictive capability compared to features derived solely from the core and entire margin (series A/B/C). This observation aligns with previous findings, where the authors conclude that a margin-based radiomic analysis better characterizes breast masses compared to analyzing only the texture information within the mass.^13^ Despite series A and B not yielding favorable outcomes, we find that the information from the core and whole margin still contributes slightly to classification in series E (eg, the 9 features in 1E including one from 1A and one from 1B). Series C, which aimed to explore the ratio between entire margin and core region features, did not yield satisfactory results. In other words, while features extracted from the tumor core provide insights into its internal composition, gradient-based features from the octant margins play a more dominant role in the overall feature set. This is further emphasized in the EFS model, where all 9 selected features are derived from the octant margins (Table S3).

A notable feature of PULSAR is its extended treatment schedule (Figure 1A), which facilitates tumor response assessment at multiple time points. Our study shows that the predictive performance of models in the E series follows the order 1E > 2E > 3E (Tables S1 and S2). In the EFS model, 5 features come from the first MRI (F1, F4, F5, F7, and F8), 3 from the second MRI (F2, F3, F9), and one from the delta mode (F6) (Table S3). These results suggest that classification is primarily driven by features derived from either first or second MRI, while delta features do not yield the most accurate predictions—somewhat contrary to our initial expectations. Nevertheless, this aligns with one study that also reported the limited prognostic value of delta features.^22^ We propose 2 possible explanations for this observation: first, tumor shrinkage during the mid-treatment phase alters the defined regions/masks, complicating the calculation of delta values; second, delta features may be influenced by pseudo-progression caused by short-term treatment-related changes, such as inflammation, vascular alterations, or radiation necrosis.

Our study has several limitations that should be acknowledged. First, the sample size of the PULSAR cohort is small and imbalanced, which affects the statistical power of analyses. Although the non-PULSAR cohort is more balanced than the PULSAR cohort, it is still limited in size. Future research should validate our approach using larger data sets. To elaborate, given the small data set, we were unable to use a fully independent data set, which might introduce potential data leakage and “over-optimistically” impact performance evaluation. While techniques like cross-validation or SVM with recursive feature elimination are effective for feature selection and model optimization, they do not completely eliminate overfitting. To help address this limitation, we employed a frequency-based feature selection approach and reduced the number of features down to 9, adhering to the guideline of maintaining a feature-to-observer ratio of at most 4:1.^23^ By focusing on features that consistently appear across samples or iterations, we filter out those likely resulting from noise or random correlations. This reduces the risk of overfitting by prioritizing stable and generalizable features and making the model less sensitive to variations in the training data set. Reducing the number of features also simplifies the model, inherently decreasing its complexity and improving its likelihood to generalize well to unseen data. To illustrate this, in Figure S1, we demonstrated that selecting the 9 most frequently occurring features resulted in a 3% reduction in ROC-AUC performance compared to merely selecting 9 features based on the entire data set. We speculate that when the model is re-evaluated on a larger data set in the future, the performance metrics summarized in Table S2 may degrade by no more than 5%, if at all.

Second, due to the small data set as well as the focus in the current phase, we used a linear-kernel SVM classifier instead of a nonlinear kernel. Further investigation is needed to further reduce redundancy among the gradient features through mutual information and variance inflation factors,^24^, ^25^, ^26^, ^27^ considering the correlation among the 3 gradient metrics selected in this study. High variance in the coefficients of SVM models may indicate multicollinearity or insufficient data.

Third, integrating gradient features with other biological and clinical factors may enhance predictive models and offer deeper insights into treatment outcomes. Our study focused solely on predicting changes in tumor volume as a surrogate endpoint for treatment response. Future research is needed to assess how changes in tumor volume correlate with local control and other important clinical outcomes. Additional clinical evidence is also required to validate the significance of a 20% GTV change as a meaningful threshold; alternative thresholds (eg, 30%, 50%) may be explored as well. Tumor necrosis may complicate tumor volume measurements. A tumor may appear to shrink due to necrosis, which does not necessarily indicate the complete eradication of viable cancer cells. Furthermore, the gradient-based method may have limited applicability to tumors with different infiltrative growth patterns, such as glioblastoma, where defining clear boundaries is challenging due to indistinct margins.

Finally, we would like to discuss the potential relevance of PULSAR and our proposed framework in the context of particle therapy (proton or carbon). While our results are based on photon therapy, we anticipate that the proposed approach can be directly applied to particle therapy as well. For both treatment modalities, PULSAR enables a more personalized and dynamic approach, continuously adapting to the patient’s specific needs (Figure 1A). Both modalities could benefit from our framework in 3 aspects: reducing feature redundancy to enhance radiomics analysis, accurately predicting treatment responses, and facilitating timely decision-making for adjusting treatment plans. Several principles of PULSAR, such as hypofractionation, adaptation, and synergy with immunotherapy, may be particularly well-suited for particle therapy due to its unique dose distribution and sensitivity to anatomical and biological changes. Specifically, the hypofractionated schedule of delivering fewer but larger doses aligns well with the higher biological effectiveness (RBE) of protons, enhancing tumor control while sparing normal tissues.^28^, ^29^, ^30^, ^31^ Proton therapy’s high sensitivity to density changes necessitates regular adaptation (eg, every 1-2 weeks) of treatment plans, which fits naturally with the fractionation scheduling of PULSAR. Additionally, PULSAR facilitates integration with other therapies, such as immunotherapy, creating opportunities for combined modalities that can expand the therapeutic window of proton therapy.^32^, ^33^

Furthermore, adaptation in proton therapy is currently limited by practical and technical challenges, such as high costs and the added expenses of adaptation protocols. PULSAR is a promising approach to overcome these challenges by reducing the number of treatment fractions, thereby lowering both overall treatment costs and time.

## Conclusion

Our study highlights the feasibility of gradient-based features for predicting treatment response for PULSAR. Gradient-based radiomics methodology, which incorporates spatial segmentation and temporal information, helps enhance prediction accuracy. The method’s demonstrated superiority over conventional radiomics techniques, along with its effectiveness in both PULSAR and non-PULSAR cohorts, underscores its potential as a unique tool for outcome prediction and decision-making. While further validation is required, these findings mark a significant step forward in developing more personalized and efficient treatment strategies, applicable to both photon and particle PULSAR.

## Data sharing statement

The data that support the findings of this study are available from the corresponding authors upon reasonable request.

## Author Contributions

Haozhao Zhang: Methodology, Software, Formal Analysis, Investigation, Writing-Original Draft, Writing-Review & Editing. Jiaqi Liu: Writing-Original Draft, Writing- Review and Editing. Michael Dohopolski, Zabi Wardak, Robert Timmerman: Conceptualization, Methodology, Supervision. Hao Peng: Methodology, Writing- Original Draft, Writing- Review and Editing, Supervision.

## Declaration of Conflicts of Interest

The authors declare that they have no known competing financial interests or personal relationships that could have appeared to influence the work reported in this paper.

## Footnotes

Appendix A Supplementary material associated with this article can be found in the online version at doi:10.1016/j.ijpt.2025.100739.
