# Supplementary material for: Gradient-Based Radiomics for Outcome Prediction and Decision-Making in PULSAR: A Preliminary Study
Source: Int J Part Ther. 2025 Feb 3;15:100739. doi: 10.1016/j.ijpt.2025.100739 (PMC11848104; doi:10.1016/j.ijpt.2025.100739)

Table S1. **Performance metrics comparison across models of PULSAR cohort. Case 1A–1E:** Models derived from 1st MRI, where 1A is based on the tumor core, 1B on the tumor margin, 1C on the core/margin ratio, 1D on octant margins, and 1E on the combination of core, margin, and octant margins. **Case 2A–2E:** Models derived from 2nd MRI datasets. **Case 3A–3E:** Models derived in delta mode, capturing temporal changes between 1st and 2nd MRI. **EFS:** Ensemble Feature Selection model, which combines features from Cases 1E, 2E, and 3E.

	Case1A	Case1B	Case1C	Case1D	Case1E	Case2A	Case2B	Case2C	Case2D	Case2E	Case3A	Case3B	Case3C	Case3D	Case3E	EFS
**Sensitivity**	0.597 ± 0.272 (95% CI: 0.563 - 0.631)	0.494 ± 0.324 (95% CI: 0.454 - 0.534)	0.599 ± 0.330 (95% CI: 0.557 - 0.640)	0.756 ± 0.253 (95% CI: 0.724 - 0.788)	0.828 ± 0.235 (95% CI: 0.799 - 0.857)	0.770 ± 0.225 (95% CI: 0.742 - 0.798)	0.607 ± 0.315 (95% CI: 0.568 - 0.647)	0.419 ± 0.284 (95% CI: 0.384 - 0.455)	0.759 ± 0.270 (95% CI: 0.726 - 0.793)	0.770 ± 0.265 (95% CI: 0.737 - 0.803)	0.501 ± 0.285 (95% CI: 0.466 - 0.537)	0.389 ± 0.306 (95% CI: 0.350 - 0.427)	0.673 ± 0.302 (95% CI: 0.635 - 0.710)	0.674 ± 0.290 (95% CI: 0.638 - 0.710)	0.779 ± 0.267 (95% CI: 0.745 - 0.812)	0.974 ± 0.104 (95% CI: 0.961 - 0.987)
**Specificity**	0.620 ± 0.130 (95% CI: 0.604 - 0.637)	0.579 ± 0.163 (95% CI: 0.559 - 0.599)	0.391 ± 0.200 (95% CI: 0.366 - 0.416)	0.793 ± 0.113 (95% CI: 0.779 - 0.808)	0.886 ± 0.102 (95% CI: 0.873 - 0.899)	0.674 ± 0.150 (95% CI: 0.655 - 0.693)	0.692 ± 0.133 (95% CI: 0.676 - 0.709)	0.623 ± 0.150 (95% CI: 0.604 - 0.642)	0.851 ± 0.123 (95% CI: 0.836 - 0.866)	0.849 ± 0.117 (95% CI: 0.835 - 0.864)	0.725 ± 0.143 (95% CI: 0.707 - 0.743)	0.656 ± 0.186 (95% CI: 0.633 - 0.680)	0.426 ± 0.148 (95% CI: 0.408 - 0.445)	0.728 ± 0.127 (95% CI: 0.712 - 0.743)	0.723 ± 0.133 (95% CI: 0.706 - 0.739)	0.973 ± 0.046 (95% CI: 0.968 - 0.979)
**Accuracy**	0.615 ± 0.108 (95% CI: 0.602 - 0.629)	0.561 ± 0.115 (95% CI: 0.547 - 0.575)	0.432 ± 0.138 (95% CI: 0.415 - 0.449)	0.786 ± 0.108 (95% CI: 0.772 - 0.799)	0.873 ± 0.087 (95% CI: 0.863 - 0.884)	0.693 ± 0.112 (95% CI: 0.679 - 0.707)	0.675 ± 0.107 (95% CI: 0.662 - 0.688)	0.582 ± 0.117 (95% CI: 0.567 - 0.596)	0.832 ± 0.100 (95% CI: 0.819 - 0.844)	0.833 ± 0.097 (95% CI: 0.820 - 0.845)	0.681 ± 0.122 (95% CI: 0.665 - 0.696)	0.602 ± 0.137 (95% CI: 0.585 - 0.619)	0.476 ± 0.116 (95% CI: 0.461 - 0.490)	0.717 ± 0.111 (95% CI: 0.703 - 0.730)	0.733 ± 0.112 (95% CI: 0.719 - 0.747)	0.974 ± 0.043 (95% CI: 0.968 - 0.979)
**AUC**	0.640 ± 0.140 (95% CI: 0.622 - 0.657)	0.587 ± 0.165 (95% CI: 0.567 - 0.608)	0.462 ± 0.155 (95% CI: 0.443 - 0.481)	0.862 ± 0.114 (95% CI: 0.848 - 0.876)	0.940 ± 0.074 (95% CI: 0.930 - 0.949)	0.724 ± 0.169 (95% CI: 0.703 - 0.746)	0.711 ± 0.166 (95% CI: 0.690 - 0.732)	0.559 ± 0.181 (95% CI: 0.536 - 0.581)	0.875 ± 0.114 (95% CI: 0.861 - 0.889)	0.869 ± 0.128 (95% CI: 0.853 - 0.885)	0.654 ± 0.181 (95% CI: 0.632 - 0.677)	0.553 ± 0.186 (95% CI: 0.530 - 0.577)	0.557 ± 0.165 (95% CI: 0.536 - 0.578)	0.763 ± 0.148 (95% CI: 0.745 - 0.782)	0.826 ± 0.111 (95% CI: 0.812 - 0.840)	0.995 ± 0.018 (95% CI: 0.993 - 0.997)
**Precision**	0.286 ± 0.135 (95% CI: 0.269 - 0.303)	0.215 ± 0.132 (95% CI: 0.198 - 0.231)	0.193 ± 0.099 (95% CI: 0.181 - 0.205)	0.510 ± 0.200 (95% CI: 0.485 - 0.535)	0.701 ± 0.223 (95% CI: 0.673 - 0.729)	0.402 ± 0.156 (95% CI: 0.382 - 0.421)	0.333 ± 0.175 (95% CI: 0.311 - 0.354)	0.212 ± 0.137 (95% CI: 0.195 - 0.229)	0.615 ± 0.243 (95% CI: 0.584 - 0.645)	0.615 ± 0.239 (95% CI: 0.586 - 0.645)	0.332 ± 0.199 (95% CI: 0.307 - 0.357)	0.216 ± 0.187 (95% CI: 0.193 - 0.240)	0.225 ± 0.093 (95% CI: 0.214 - 0.237)	0.396 ± 0.168 (95% CI: 0.375 - 0.417)	0.434 ± 0.186 (95% CI: 0.411 - 0.457)	0.922 ± 0.134 (95% CI: 0.905 - 0.938)
**F1 Score**	0.377 ± 0.160 (95% CI: 0.357 - 0.397)	0.289 ± 0.172 (95% CI: 0.268 - 0.311)	0.283 ± 0.137 (95% CI: 0.266 - 0.300)	0.590 ± 0.191 (95% CI: 0.566 - 0.614)	0.727 ± 0.180 (95% CI: 0.705 - 0.750)	0.508 ± 0.137 (95% CI: 0.491 - 0.525)	0.415 ± 0.198 (95% CI: 0.390 - 0.439)	0.273 ± 0.169 (95% CI: 0.252 - 0.294)	0.643 ± 0.201 (95% CI: 0.618 - 0.668)	0.649 ± 0.196 (95% CI: 0.625 - 0.674)	0.382 ± 0.201 (95% CI: 0.357 - 0.407)	0.259 ± 0.190 (95% CI: 0.236 - 0.283)	0.332 ± 0.134 (95% CI: 0.315 - 0.349)	0.483 ± 0.187 (95% CI: 0.460 - 0.506)	0.541 ± 0.187 (95% CI: 0.517 - 0.564)	0.940 ± 0.103 (95% CI: 0.927 - 0.952)

Table S2: P-Value matrix for cross-model evaluation of PULSAR cohort. The table presents the pairwise statistical comparisons of performance metrics between individual models and the EFS model. The metrics include sensitivity, specificity, accuracy, AUC, precision, and F1-score. Each row represents the p-values obtained for pairwise comparisons. Statistical significance is assessed using Welch's t-test with Bonferroni correction. p-values < 0.002 is considered statistically significant and marked with an asterisk (*).

	Case 1D vs. Case 1E	Case 1D vs. Case 2D	Case 1D vs. Case 2E	Case 1D vs. Case 3D	Case 1D vs. Case 3E	Case 1D vs. EFS	Case 1E vs. Case 2D	Case 1E vs. Case 2E	Case 1E vs. Case 3D	Case 1E vs. Case 3E	Case 1E vs. EFS	Case 2D vs. Case 2E	Case 2D vs. Case 3D	Case 2D vs. Case 3E	Case 2D vs. EFS	Case 2E vs. Case 3D	Case 2E vs. Case 3E	Case 2E vs. EFS	Case 3D vs. Case 3E	Case 3D vs. EFS	Case 3E vs. EFS
**Sensitivity**	< 0.002*	**0.887**	**0.547**	< 0.002*	**0.332**	< 0.002*	**0.003**	**0.010**	< 0.002*	**0.029**	< 0.002*	**0.656**	< 0.002*	**0.422**	< 0.002*	< 0.002*	**0.716**	< 0.002*	< 0.002*	< 0.002*	< 0.002*
**Specificity**	< 0.002*	< 0.002*	< 0.002*	< 0.002*	< 0.002*	< 0.002*	< 0.002*	< 0.002*	< 0.002*	< 0.002*	< 0.002*	**0.892**	< 0.002*	< 0.002*	< 0.002*	< 0.002*	< 0.002*	< 0.002*	**0.663**	< 0.002*	< 0.002*
**Accuracy**	< 0.002*	< 0.002*	< 0.002*	< 0.002*	< 0.002*	< 0.002*	< 0.002*	< 0.002*	< 0.002*	< 0.002*	< 0.002*	**0.915**	< 0.002*	< 0.002*	< 0.002*	< 0.002*	< 0.002*	< 0.002*	**0.094**	< 0.002*	< 0.002*
**AUC**	< 0.002*	**0.210**	**0.502**	< 0.002*	< 0.002*	< 0.002*	< 0.002*	< 0.002*	< 0.002*	< 0.002*	< 0.002*	**0.611**	< 0.002*	< 0.002*	< 0.002*	< 0.002*	< 0.002*	< 0.002*	< 0.002*	< 0.002*	< 0.002*
**Precision**	< 0.002*	< 0.002*	< 0.002*	< 0.002*	< 0.002*	< 0.002*	< 0.002*	< 0.002*	< 0.002*	< 0.002*	< 0.002*	**0.969**	< 0.002*	< 0.002*	< 0.002*	< 0.002*	< 0.002*	< 0.002*	**0.018**	< 0.002*	< 0.002*
**F1 Score**	< 0.002*	**0.003**	< 0.002*	< 0.002*	**0.004**	< 0.002*	< 0.002*	< 0.002*	< 0.002*	< 0.002*	< 0.002*	**0.728**	< 0.002*	< 0.002*	< 0.002*	< 0.002*	< 0.002*	< 0.002*	< 0.002*	< 0.002*	< 0.002*

Table S3 : Summary of the 9 selected features in the EFS model. Region: the region from which the feature was derived from $\{M_{core}, M_{margin}, M_i, M_2 \ldots M_8\}$(see Fig. 2). Gradient Parameter: the gradient parameter used to compute the feature map, including gradient magnitude (GM), radial gradient (RG), and radial deviation (RD). Statistical Metric: the metric associated with the feature value, including mean, standard deviation (STD), and coefficient of variation (CV). Feature Source: the dataset from which the feature was derived, case 1E, case 2E, or case 3E. Feature Weight Coefficient: coefficient values of the nine selected features. Feature Values (Z-score): z-scores (mean ± standard deviation) of the feature values for two groups. P-value: significance comparison of feature values using either the t-test or Mann-Whitney U test (marked with *).

Feature	Region	Gradient parameter	Statistical metric	Feature source	Feature Weight Coefficient	Feature Values (Z-score)		
						Non-responder	Responder	P-value
F1	M_6	RG	STD	1E	-1.261	-0.527 ± 0.265	0.134 ± 1.071	0.015*
F2	M_6	RG	Mean	2E	-1.054	-0.587 ± 0.312	0.149 ± 1.058	0.001*
F3	M_6	RD	CV	2E	-1.004	-0.767 ± 0.727	0.195 ± 0.965	0.001#
F4	M_6	GM	Mean	1E	-0.694	-0.433 ± 0.551	0.110 ± 1.057	0.133
F5	M_4	RG	Mean	1E	0.486	0.911 ± 1.542	-0.232 ± 0.620	0.003*
F6	M_1	RD	STD	3E	0.774	0.135 ± 0.712	-0.034 ± 0.838	0.496
F7	M_4	RD	Mean	1E	0.899	0.166 ± 0.607	-0.042 ± 1.073	0.760
F8	M_2	RG	CV	1E	1.008	0.550 ± 0.967	-0.140 ± 0.959	0.030*
F9	M_8	RG	CV	2E	1.229	0.467 ± 1.349	-0.119 ± 0.849	0.085

Table S4. **Performance metrics comparison for the non-PULSAR cohort between the gradient model and standard radiomics model.** Values are presented as mean ± standard deviation with 95% confidence intervals (CI) in parentheses.

	Gradient Model	Radiomics Model
**Sensitivity**	0.921 ± 0.135 (95% CI: 0.904 - 0.938)	0.772 ± 0.224 (95% CI: 0.744 - 0.800)
**Specificity**	0.832 ± 0.190 (95% CI: 0.809 - 0.856)	0.713 ± 0.213 (95% CI: 0.687 - 0.740)
**Accuracy**	0.875 ± 0.115 (95% CI: 0.860 - 0.889)	0.741 ± 0.145 (95% CI: 0.723 - 0.760)
**AUC**	0.933 ± 0.104 (95% CI: 0.920 - 0.946)	0.839 ± 0.145 (95% CI: 0.821 - 0.857)
**Precision**	0.862 ± 0.149 (95% CI: 0.843 - 0.880)	0.735 ± 0.180 (95% CI: 0.713 - 0.757)
**F1 Score**	0.879 ± 0.112 (95% CI: 0.865 - 0.893)	0.732 ± 0.164 (95% CI: 0.712 - 0.753)

Figure S1. Illustration of the frequency-based feature selection method. Stratified 5-fold cross-validation (50 iterations) was employed, along with frequency-based feature selection, to help mitigate potential overfitting and bias. Feature extraction for case 1E was selected as an example to demonstrate how the top 9 most frequent features were identified. ROC-AUC are compared with non-frequency-based model (entire dataset and 9 features), showing a reduction of 3%. The seven overlapping features out of nine between frequency-based and non-frequency-based methods are highlighted in the red boxes.

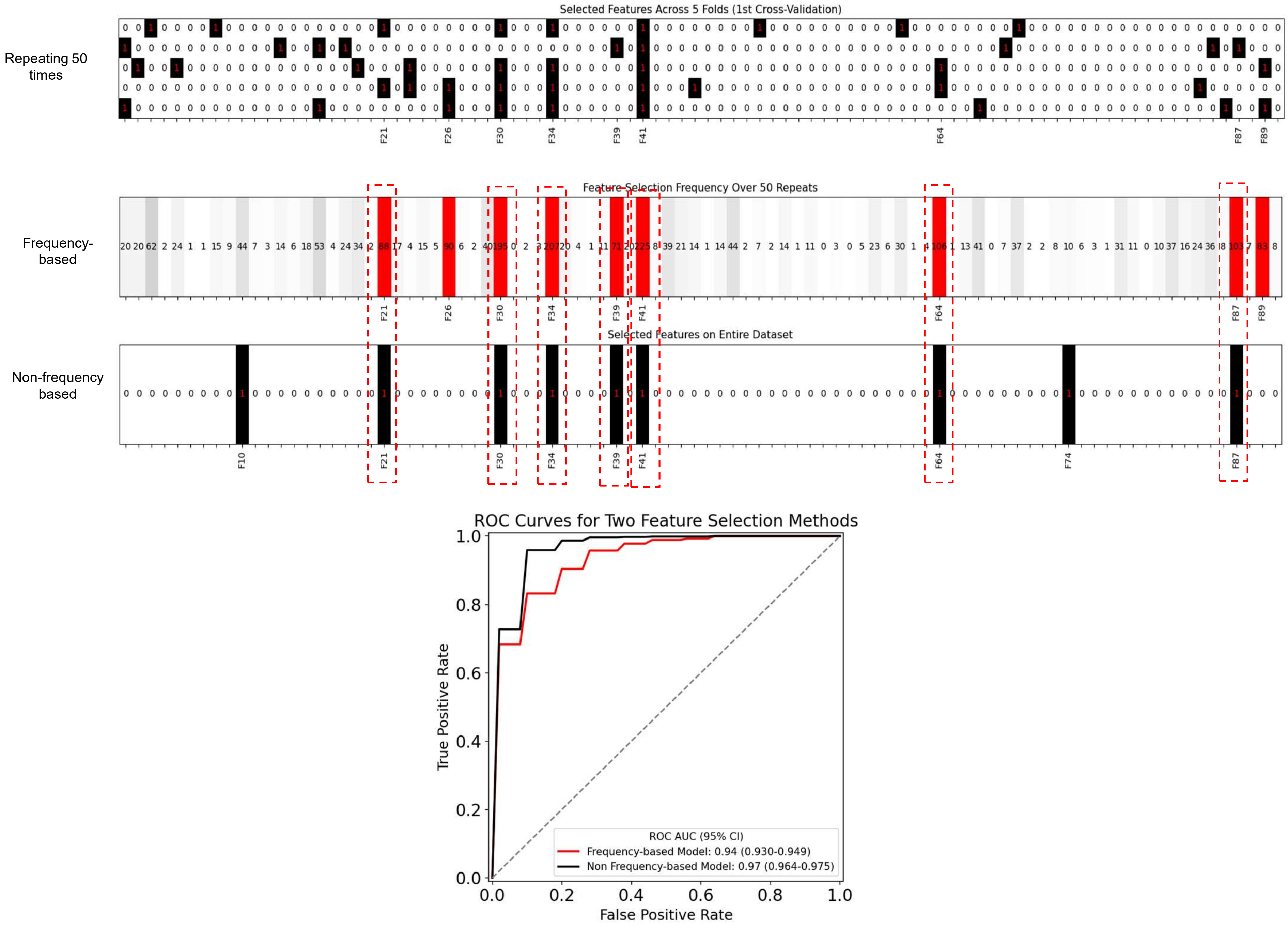

Supplement: Supplementary file 1 — Supplementary material [file mmc1.pdf]
